# Chronic Cocaine Abuse as a Cause of Sinus Bradycardia

**DOI:** 10.7759/cureus.37524

**Published:** 2023-04-13

**Authors:** Aliaa Mousa, Muhammad Humayoun Rashid, Syeda Neelam Yamin Bukhari, Mohammad Abu-Abaa, karan h pahuja

**Affiliations:** 1 Internal Medicine, Capital Health Regional Medical Center, Trenton, USA; 2 Internal Medicine, Nishtar Medical University, Multan, PAK

**Keywords:** sinus, stroke, bradycardia, chronic, cocaine

## Abstract

Cocaine abuse has an overwhelming effect on the healthcare system due to its multiple complications. Cardiovascular complications carry the highest burden. Common cardiovascular manifestations of cocaine are related to its adrenergic effects due to the inhibition of dopamine and norepinephrine uptake at the postsynaptic terminal. However, chronic abuse can lead to desensitization of adrenergic receptors, which can lead to bradycardia. Sinus bradycardia can be one of the markers of chronic cocaine abuse, as exemplified in this case report. Therefore clinicians should be aware of this association.

## Introduction

Cocaine has an overwhelming effect on the healthcare system because of its neurologic, gastrointestinal, renal, and endocrine complications; however, its cardiovascular effects are the most common [1]. The effects of cocaine use place a substantial economic and social burden on the healthcare system. Approximately 5 to 10% of emergency department visits in the United States are thought to be related to cocaine use [2]. Patients who abuse cocaine risk life-threatening consequences, including tach dysrhythmia, severe hypertension, acute coronary syndrome, stroke, acute myocardial infarction and renal failure, seizure, hyperthermia, cocaine-induced rhabdomyolysis, and fetal/maternal morbidity and mortality [3]. Common cardiovascular manifestations of cocaine are related to its adrenergic effects due to the inhibition of dopamine and norepinephrine uptake at the postsynaptic terminal [4]. Some studies have reported that bradycardia is also related to regular cocaine usage [5,6], which is in contrast to the tachycardia and high blood pressure that is more commonly reported. This could increase the chances of missing this finding because of its rare presentation. Here we will discuss an individual that presented with a pre-hospital stroke alert and was found to have an acute ischemic stroke and cocaine-induced bradycardia.

## Case presentation

The patient was a 63-year-old female with a past medical history of hypertension who presented to the emergency department with acute onset left-sided weakness, left facial droop, and slurring of speech. On examination, her BP was 172/93 mmHg, HR 48/min, RR 18/min, and afebrile 37.2°C. She was found to have reduced motor strength in the left upper and lower limbs and left homonymous hemianopia. CT scan of her head showed an acute infarction in the medial right occipital lobe and the medial posterior temporal lobe. The findings were later confirmed with a brain MRI scan. A CT angiogram of her head and neck showed narrowing of the right posterior cerebral artery (PCA) in the P1 and P2 segments, which corresponded to the area of infarct. The patient presented outside the thrombolysis window (greater than 4.5 hours); therefore, a decision was made against it. She was given aspirin 325mg and then started dual antiplatelet therapy with aspirin 81mg and Clopidogrel 75mg. Atorvastatin 80 mg was also added for secondary prevention. Permissive hypertension was allowed for 48 hours to improve blood flow to the brain. 

**Figure 1:**
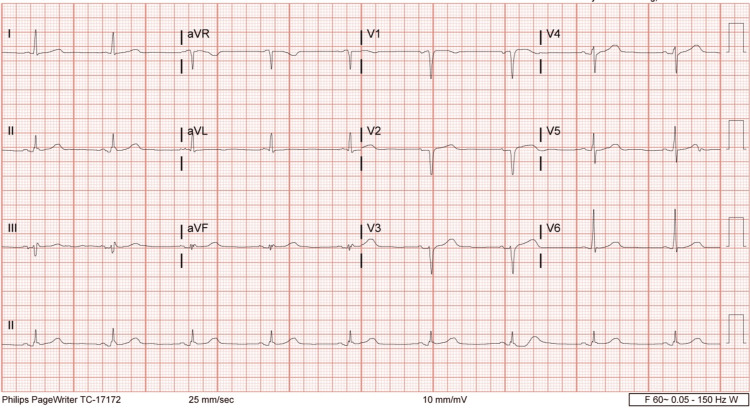
Electrocardiogram

The patient was admitted to the stroke unit with telemetry monitoring and NIH stroke scale assessment every 12 hours. Further workup to rule out the possible causes and risk factors of her stroke was done, including basic lab workup (Table 1), electrocardiogram (EKG), hemoglobin A1C (HbA1C), lipid panel, thyroid stimulating hormone (TSH), echocardiography, and urine drug screen. HbA1C was 5.6 which ruled out diabetes. Her total Cholesterol level 204 mg/dL and a LDL level of 145 mg/dL. Thyroid-stimulating hormone (TSH) was 2.5 mIU/L (normal values are 0.5 to 5.0 mIU/L). The urine drug screen was positive for cocaine. EKG showed sinus bradycardia with a heart rate of 54 beats per minute (Figure 1). Echocardiography revealed no heart thrombus, patent foramen ovale (PFO), wall motion abnormality, and a normal ejection fraction; therefore, cardioembolism was considered a less likely cause. This patient's underlying risk factors for stroke were hypertension, hyperlipidemia, and cocaine abuse. At this point, no particular cause of her bradycardia could be found. The patient was neither an athlete nor on any heart rate-controlling medication. The patient expressed that she has been using cocaine for more than 10 years, as she used to sprinkle cocaine on her daily smoked marijuana cigarettes. She further reported that when she started using cocaine, her heart rate at that time was very high but gradually became lower over time. During the first week of her hospital stay, she did not develop any withdrawal symptoms; her heart rate remained within the range of the 40s and without any symptoms, but then gradually increased to the 80s until she was discharged. Physical therapy was done for her ataxia and weakness. She was transferred to an inpatient rehabilitation center for continued care, where she regained some of her muscle strength. The patient was advised to continue taking aspirin and Clopidogrel for 90 days and then continue aspirin and statin lifelong for secondary prevention of a potential future stroke. At discharge, the patient was educated about the complications of cocaine abuse and was counseled against its use.

**Table 1:** Table 1 (Basic laboratory workup).

Tests	Results	Normal ranges
White blood cell count (WBC)	11.58 x10^3/mcL	4 to 10 x10^3/mcL
Hemoglobin	16.4 g/dL	11.2 to 15.7 g/dL
Red blood cell count (RBC)	5.33 x10^6/mcL	3.9 to 5.2 x10^6/mcL
Platelets	309 x10^3/mcL	150 to 400 x10^3/mcL
Sodium	140 mmol/L	137 to 145 mmol/L
Potassium	4.0 mmol/L	3.5 to 5.1 mmol/L
Chloride	106 mmol/L	98 to 107 mmol/L
Bicarbonate	25 mmol/L	22 to 30 mmol/L
Serum creatinine	0.89 mg/dl	0.52 to 1.04 mg/dl
Blood urea nitrogen (BUN)	15 mg/dl	7 to 17 mg/dl
Alkaline phosphatase (ALP)	106 U/L	38 to 126 U/L
Aspartate transaminase (AST)	34 U/L	14 to 36 U/L
Alanine transaminase (ALT)	23 U/L	0 to 34 U/L
Albumin	4.2 g/dl	3.5 to 5.0 g/dl
Total cholesterol	204 mg/dl	51 to 200 mg/dl
High density lipoprotein (HDL)	31.6 mg/dl	40 to 60 mg/dl
Low density lipoprotein (LDL)	145 mg/dl	0 to 100 mg/dl
Triglycerides	136 mg/dl	0 to 150 mg/dl

## Discussion

The purpose of this case presentation is to highlight that bradycardia can be related to the regular usage of cocaine. Cocaine-induced bradycardia has not been frequently reported but has been reported in human studies. In one human observational study, Franklin et al. assessed sinus bradycardia in chronic cocaine users. The study compared 162 patients with a history of cocaine use to 149 non-cocaine users in which, age and gender-matched controls [5]. He reported habitual cocaine use was an independent predictor of sinus bradycardia and was associated with a 7-fold increase in developing sinus bradycardia (95% CI 2.52 to 19.74, p = 0.0002).

In another human study, Jyoti Sharma et al. compared the heart rates of 97 cocaine-dependent individuals to 8513 control subjects [6]. The study revealed an increase in developing bradycardia in cocaine-dependent patients than in non-cocaine users, with the odds of 3.02 for bradycardia and 5.11 for severe bradycardia.

Another researcher C J Bruce observed that trauma patients with relative bradycardia after acute blood loss had more underlying cocaine exposure than those with tachycardia. He then experimented on rats and gave intraperitoneal cocaine 20 mg/kg/day for 14 days versus saline infusion. He discovered that cocaine-treated rats had more relative bradycardia and a more significant drop in mean arterial pressure within 5 minutes of hemorrhagic shock [7].

The suggested mechanism is that chronic cocaine abuse can lead to desensitization of beta-adrenergic receptors secondary to continuous exposure to adrenergic neurotransmitters, producing slower heart rates [8]. Ramirez FD et al. suggested another mechanism of action, habitual cocaine abuse can cause diffuse myocardial necrosis and myopathy involving the conduction system producing sinus bradycardia [9]. The effect is the opposite during acute use of cocaine, which leads to reduced uptake of neurotransmitters at the postsynaptic membrane, increasing its concentration in synaptic cleft which can cause an enhanced sympathomimetic response, leading to tachycardia and high blood pressure [4].

In the case discussed above, the patient was hospitalized for 14 days, and in the second week, her heart rate gradually started to increase, which, eventually in the 80s at the time of discharge. This finding corresponds to a prior study [10] showing that some cocaine-dependent subjects demonstrated an increase in resting heart rate over time during their hospitalization after abstaining from cocaine [10]. This suggests that cocaine-induced bradycardia is reversible, and cocaine abstinence can lead to its resolution, which might be a marker of long-term cocaine abstinence.

## Conclusions

Cocaine abuse can present as sinus bradycardia which can be associated with the duration of cocaine abuse. Chronic exposure can lead to desensitization of adrenergic receptors, causing a decrease in heart rate, as evident from the case presented and discussed in this report.
